# Patient experiences with and adherence to Crohn’s disease exclusion diet in Dutch Crohn’s disease patients: a cohort study

**DOI:** 10.1177/17562848251323553

**Published:** 2025-03-12

**Authors:** Fleur T. R. Wijers, Suzanne M. C. van Zundert, Charlotte M. Verburgt, Nikki van der Kruk, Johan E. Van Limbergen, Nicolette J. Wierdsma

**Affiliations:** Department of Nutrition and Dietetics, Amsterdam University Medical Centers, Meibergdreef 9, 1105 AZ Amsterdam, The Netherlands; Department of Nutrition and Dietetics, Amsterdam University Medical Centers, Emma Children’s Hospital, Meibergdreef 9, 1105 AZ Amsterdam, The Netherlands; Department of Pediatric Gastroenterology, Amsterdam University Medical Centers, Emma Children’s Hospital, Amsterdam, The Netherlands; Department of Pediatric Gastroenterology, Amsterdam University Medical Centers, Emma Children’s Hospital, Amsterdam, The Netherlands; Department of Pediatric Gastroenterology, Amsterdam University Medical Centers, Emma Children’s Hospital, Amsterdam, The Netherlands; Research Institute Amsterdam Gastroenterology Endocrinology Metabolism, Amsterdam, The Netherlands; Tytgat Institute for Liver and Intestinal Research, Amsterdam, The Netherlands; Department of Nutrition and Dietetics, Amsterdam University Medical Centers, Amsterdam, The Netherlands; Research Institute Amsterdam Gastroenterology Endocrinology Metabolism, Amsterdam, The Netherlands

**Keywords:** adherence, CDED, compliance, Crohn’s disease, Crohn’s disease exclusion diet, diet, IBD, nutritional therapy, partial enteral nutrition, patient experiences

## Abstract

**Background:**

Dietary therapy is commonly used as a treatment for Crohn’s disease (CD). High dietary adherence is associated with achieving clinical remission. Crohn’s disease exclusion diet (CDED) is a relatively new therapy in the management of CD.

**Objective:**

This publication aims to assess the first real-life patient experience with and adherence to Crohn’s disease exclusion diet plus partial enteral nutrition (CDED + PEN) in Dutch children and adults with mild-to-moderate CD.

**Design:**

Interviews were performed with patients and/or caregivers prospectively after phases I, II, and III, and once after finishing therapy in a retrospective cohort.

**Methods:**

We obtained data on patient experiences with CDED and the accompanying Modulife patient support platform and assessed effectiveness from patients’ and physicians’ perspectives based on medical and clinical data obtained from the patient file. The interview contained open questions, 5-point Likert scales, and Net Promotor Scores (NPS).

**Results:**

Sixty-nine patients were included (52 pediatric and 17 adults). Approximately half of the patients in the prospective cohort and the majority (83%) of patients in the retrospective cohort would recommend CDED to others. Two-thirds of the patients would reconsider starting CDED again. A positive NPS (31) was given for recommending the support platform to others with the recipes feature as the most used and esteemed part. Median fecal calprotectin and C-reactive protein gradually decreased from baseline to 18 weeks of therapy in both children and adults. Two-thirds of the physicians assessed the diet as showing good effectiveness and would continue the dietary therapy at each phase of the diet.

**Conclusion:**

Many mild-to-moderate active CD patients may experience positive outcomes and have good experiences with the CDED + PEN dietary therapy and the associated Modulife patient support platform. This study might add valuable patient perspectives to the growing clinical use of CDED in managing CD.

## Introduction

The incidence of inflammatory bowel diseases (IBD), including Crohn’s disease (CD), is increasing.^1^ CD negatively affects health, reduces quality of life, and increases healthcare costs.^2^ Diet significantly impacts human health. The Western diet, characterized by high intake of saturated fats and sugars and low fiber, contains components with pro-inflammatory effects, influencing the microbiota composition, which is a factor in IBD etiology.^3,4^

Dietary therapy has become the first-line treatment for children with mild-to-moderate CD to induce remission and later for adults to avoid corticosteroids or additional immunosuppression, according to guidelines.^5–8^ Evidence supports lifestyle and dietary changes as potential inflammatory modulators, reducing the need for immunomodulatory drugs.^5^ Exclusive enteral nutrition (EEN), the most commonly used dietary therapy, involves a nutritionally balanced liquid meal replacement that excludes all solid foods and beverages, typically for 6–8 weeks.^5^ However, EEN presents challenges for both patients and families due to the absence of solid food during treatment.^9^ EEN significantly alters family dynamics, affects mealtimes, and is difficult to tolerate due to poor palatability, dietary monotony, social impact, and integration into school and/or work activities.^10–13^ Sometimes, it must be administered via a nasogastric tube, impacting healthcare provision and coping mechanisms with a life-altering disease.^14^ EEN is mainly studied as a short-term treatment for inducing remission, while partial enteral nutrition (PEN) for long-term maintenance of remission is rarely used and varies across centers.^9,10^

A novel dietary therapy, the Crohn’s disease exclusion diet (CDED) plus PEN, was developed: a whole-food diet designed to reduce exposure to potentially pro-inflammatory dietary components that might increase inflammation by negatively affecting the microbiome and intestinal barrier.^10,15,16^ CDED plus PEN is as effective as EEN in achieving clinical and biochemical remission but superior in tolerance and compliance in pediatric CD patients.^15,17^ While data on the use of dietary therapy in adults are still limited, studies show promising results.^18^ Recently, CDED has been added to guidelines for adults as a treatment option.^7,13,18–21^

EEN has been proven effective in inducing remission in both adults and children with CD, with meta-analyses supporting its use as a first-line treatment.^12,22^ Similarly, CDED has shown positive effects on clinical remission, though its long-term efficacy is less extensively studied than EEN.^15,18,23^

Both EEN and CDED + PEN face challenges in adherence and acceptance. EEN, being a liquid diet, struggles with issues related to socialization, taste, and practicality, leading to lower adherence.^24^ By contrast, CDED + PEN offers better tolerance and higher adherence rates, making it a promising alternative for inducing remission in both children and adults with CD.^25^ It is crucial to compare these therapies in terms of patient acceptance and quality of life.

CDED + PEN provides both a short-term strategy for induction and a long-term approach for maintaining remission. It incorporates whole foods, making it more palatable and better tolerated than EEN over time. Challenges include fear of trying new foods, time commitment for meal preparation, adapting to different settings (such as school, work, or holidays), and concerns about the cost of fresh foods.^10^

The CD has an unpredictable course and often requires complex medication regimens, making adherence challenging, especially in pediatric patients.^26,27^ Poor adherence to oral medications is linked to factors related to the illness, the child, and their family. Maintaining long-term dietary therapy is also difficult.^17^ This has led to interest in developing better-tolerated dietary strategies. Diet adherence is influenced by factors such as education, support, patient characteristics, access to food, and mealtime preparation, requiring active patient engagement, responsibility, and lifestyle changes.^28,29^

Intervention strategies are needed to improve adherence, as higher adherence to dietary therapies like EEN and CDED is linked to achieving clinical remission.^15,30^ Dietitians play a key role in supporting adherence through dietary guidance.^18,30–34^ However, data on patients’ experiences with the CDED, especially as an add-on therapy, are scarce.^31^ Understanding the patient’s perspective is crucial for improving adherence and clinical outcomes. It is hypothesized that patients who complete the first two phases of therapy will be satisfied with the treatment.

The study aimed to evaluate real-life experiences and adherence to CDED + PEN therapy and the Modulife support platform in Dutch children and adults with mild-to-moderate CD. A secondary aim was to assess the therapy’s effectiveness from both physician and patient perspectives.

## Methods

### Design

Both a retrospective and a prospective cohort were established to collect data. In the retrospective cohort, a 30-min structured qualitative telephone interview was conducted with patients who followed the CDED + PEN from September 2019 to June 2022 without altering their medication, by an independent dietician or physician (not by the patient’s clinician). In addition, for children under the age of 16, the interview was conducted with both the child and the caregiver. For those aged 16 and older, the interview was conducted with the adolescent, and, if they wished, also with the parents. Prospective data were collected between August 2021 and February 2023 during scheduled dietetic consultations at the outpatient clinic as part of the regular dietary treatment at the start of CDED (baseline), at the end of phase I (W6) and phase II (W12), and 6 weeks after starting phase III (W18). Subsequently, standard dietary care continued if necessary. All patients received dietary guidance and instructions regarding phases I, II, and III of CDED + PEN from a dedicated dietician as part of the medical treatment.^3,15,19,32^ The Medical Ethics Review Committee determined that both study cohorts were not subject to Medical Research Involving Human Subjects Acts (WMO). Written informed consent was obtained from all patients and/or their responsible parents. The results of this study were reported in accordance with the STROBE (Strengthening the Reporting of Observational Studies in Epidemiology) guidelines^35^ (see Supplemental Table 2).

### Crohn’s disease exclusion diet

The principles of the CDED have been described elsewhere.^16,19,32^ Briefly, it is a whole-food diet with additional PEN, designed to reduce exposure to dietary components that negatively affect the microbiome, intestinal barrier, immunity, and inflammation by limiting animal fat, wheat, red meat, taurine-rich products, ultra-processed foods, emulsifiers, carrageenan, sulfites, and artificial sweeteners. On the other hand, the diet is rich in fruit and vegetable fibers, resistant starch (which are needed to produce short-chain fatty acids), and lean high-quality proteins to ensure a balanced diet. During the different phases of the diet, whole foods are gradually reintroduced. CDED + PEN therapy included full access to the Modulife patient support platform (website or app version), which provided online access for asking questions to experts, infographics with dietary information, weekly menus, and phase-specific recipes.

### Inclusion criteria

Patients were included in one of the two cohorts if they had new-onset or existing mild-to-moderate active CD, treated with CDED + PEN as dietary therapy as part of their medical treatment at the departments of (pediatric) gastroenterology at the Amsterdam University Medical Center. Patients with severe disease activity or those on a strict vegan diet were excluded from the studies. To minimize bias, all consecutive CD patients treated with CDED were approached for the prospective cohort during the aforementioned period. For the recruitment of patients in the retrospective cohort, all patients previously treated with CDED + PEN in the period preceding inclusion for the prospective cohort were sent a letter with study information and a refuse-to-participate response form, which could be returned if applicable. Subsequently, all willing patients were contacted for the interview.

### Data collection

#### Demographics

Data were collected as reported by the treating physician or dietician from the medical electronic patient files at baseline and at the end of phases I, II, and 6 weeks after phase III. Demographic data included, among other things, gender, date of birth, and anthropometric data (height, weight, body mass index (BMI), and SD scores for pediatric patients). Clinical data included applied dietary therapy in medical history, medical information (such as date of diagnosis, magnetic resonance imaging (MRI) results at diagnosis, induction therapies before CDED, and maintenance medication at the start of CDED). Symptoms including abdominal pain, defecation pattern (frequency and consistency, blood, and/or mucus admixture), urge, general well-being, and other symptoms and complications were also collected. In addition, laboratory values including fecal calprotectin (FCP; µg/g), C-reactive protein (CRP) (mg/L), and blood sediment (BSE) (mm/h) were obtained.

#### CDED + PEN experiences

In both the retrospective and prospective parts of this study, patients were asked about their experience with CDED (feasibility, impact on daily life, recommendation of the therapy to others, and whether they would consider following the diet again in the future if necessary), as well as their use of the Modulife patient support platform (whether it had been used, which parts and usefulness of the app/platform features) and the taste of the prescribed PEN (Modulen IBD^®^) or the use of other oral nutritional supplements, if applicable. For retrospective data collection, a telephone interview with a trained dietician or physician, lasting approximately 30 min, was conducted to assess patients’ adherence to and experiences with the different phases of the diet. Responses were scored using the Net Promotor Score (NPS, range −100 until +100), yes/no answer options, 5-point Likert scales (1 = strongly agree/always to 5 = strongly disagree/never), and open-ended questions for opinions, experiences, and feedback.

#### CDED compliance

In both cohorts, cessation of CDED + PEN dietary therapy, either at the patient’s wishes or the doctor’s advice, was labeled as a dropout. In the retrospective cohort, compliance was assessed by asking patients how often they consumed prohibited foods or cheat products per week during the three phases. If patients ate more than 1 cheat product/meal per week in phases I and II, it was considered poor/non-compliant. If they ate more than 2 cheat products/meals per week in phase III, it was also considered as poor/non-compliant. In addition, data were collected about whether patients continued the diet after each phase. In the prospective cohort, compliance was assessed at W6, W12, and W18 using a Modified Medication Adherence Report Scale (MARS) questionnaire,^36^ containing 11 questions for a total score of 13 points (see Supplemental Table 1). A score of >10 points was considered good compliance, 7–10 points as moderate compliance, and <6 points as poor/non-compliance. Question 1 was a subjective adherence question, worth 2 points for the answer “very often/always,” 1 point for other answers, and 0 points for “rarely/never.” Questions 2–10 each were each worth 1 point (answer yes/no) and finally question 11 on the use of prescribed PEN (Modulen IBD) was rated from 0 to 2 points.^15,30,36^

#### Effectiveness (physicians and patients’ perspective)

To assess the effect of CDED + PEN from a physician’s perspective, the treating physicians were asked to evaluate the effectiveness of the dietary therapy retrospectively, based on medical data (FCP, CRP, BSE, MRI results, and medication use), and clinical data (symptoms, defecation) for each phase, using the following three items:

1. Effect of CDED on symptoms (no effect, moderate effect, and good to very good effect).
2. Indication to continue with the dietary therapy (yes, no, doubt).
3. Indication to start another additional therapy (yes, no, doubt).

To assess the effect of the dietary therapy from the patient’s perspective, patients were asked to score the effectiveness of the CDED + PEN dietary therapy on their symptoms using a scale from 0 to 10. Scores of 6 or higher (from the patient) or rated as moderate or good to very good effect (by the physician) were considered as a “good effect.”

### Statistical analysis

Data were collected in Electronic Data Capture Castor v2022.5.2.0 on the Amsterdam UMC secured server. Statistical analyses were performed using Excel v2016.1.1 and IBM SPSS v28.01.1. The Wilcoxon signed-rank test was used for changes in continuous data over time (FCP and CRP). The chi-squared test was used to test for significant differences between pediatric and adult patients (CDED as monotherapy or add-on therapy). Missing data were recorded, and loss to follow-up was addressed as a dropout.

## Results

### Demographics

A total of 26 prospective patients and 94 retrospective cohort patients were approached. All patients in the prospective cohort and 43 in the retrospective cohort agreed to participate. Approximately half of the approached patients were not included in the retrospective cohort (2 patients were excluded because they did not follow the CDED diet with whole foods, 28 patients returned the refuse-to-participate letter, and 21 patients could not be reached after multiple calls). In total, 69 patients were included, consisting of 52 pediatric and 17 adult patients.

Patient characteristics are depicted in Table 1. Most pediatric patients started CDED therapy at diagnosis (71%) and on the physician’s initiative (94%), in contrast to adult patients in whom the majority started dietary therapy several years after diagnosis (94%) and on their initiative (47%).

**Table 1.** Patient characteristics and demographic data at the start of CDED + PEN therapy (both cohorts).

Characteristics and demographics	Pediatric (*N* = 52)	Adult (*N* = 17)	Total (*N* = 69)
Gender, female (*N* (%))	27 (52)	14 (82)	41 (59)
Age at diagnosis in years (median (IQR))	13 (10–15)	33 (20–49)	15 (11–17)
Age in years at start CDED (median (IQR))	15 (13–16)	46 (33–56)	16 (14–18)
Started CDED after diagnosis (*N* (%))*	37 (71)	1 (6)	38 (55)
Initiative to start CDED (*N* (%))**			
Physician	49 (94)	7 (41)	56 (81)
Dietician	1 (2)	2 (12)	3 (4)
Patient	2 (4)	8 (47)	10 (14)
BMI at start CDED (*N* (%))	Non applicable		Non applicable
<18.5 kg/m^2^		1 (6)	
⩾18.5–24.9 kg/m^2^		9 (53)	
⩾25.0–29.9 kg/m^2^		7 (41)	
Weight for height (*Z*-score) at start CDED (*N* = 49) (*N* (%))		Non applicable	Non applicable
<−2.0	5 (12)		
−2.0 ↔ +2.0	39 (78)		
>2.0 SD	5 (10)		
Use of co-medication at the start of CDED, (*N* (%))***			
CDED as monotherapy	30 (63)	3 (18)	33 (51)
CDED with medication as induction	8 (16)	5 (29)	13 (20)
CDED with medication as maintenance	10 (21)	9 (53)	19 (29)
Prednisone	0 (0)	0 (0)	0 (0)
Entocort	0 (0)	0 (0)	0 (0)
Thiopurine	3 (6)	0 (0)	3 (3)
MTX	1 (2)	2 (12)	3 (3)
5-ASA	1 (2)	0 (0)	1 (2)
Biologics	6 (12)	7 (41)	13 (20)
Other	5 (10)	3 (18)	8 (12)
Combination	2 (4)	2 (12)	4 (6)

* *p* < 0.01, the difference between the number of pediatric and adult patients starting CDED after diagnosis or later.

** *p* < 0.01, the difference between the number of pediatric and adult patients starting CDED on the physician’s, dietician’s, or patient’ initiative.

*** *p* < 0.05, the difference between the number of pediatric and adult patients receiving CDED as monotherapy or combination therapy.

BMI, body mass index; CDED, Crohn’s disease exclusion diet; IQR, interquartile range; MTX, methotrexate; PEN, partial enteral nutrition.

Two-thirds of the pediatric patients (63%) received CDED + PEN as monotherapy, compared to 18% in adults (*p* < 0.05), with CDED most often prescribed as add-on therapy in adults (41% biologics). In terms of anthropometric data, it was noted that most of the pediatric and adult patients had a normal *Z*-score or BMI at the start of dietary therapy.

### CDED + PEN experience

**Figure 1.**
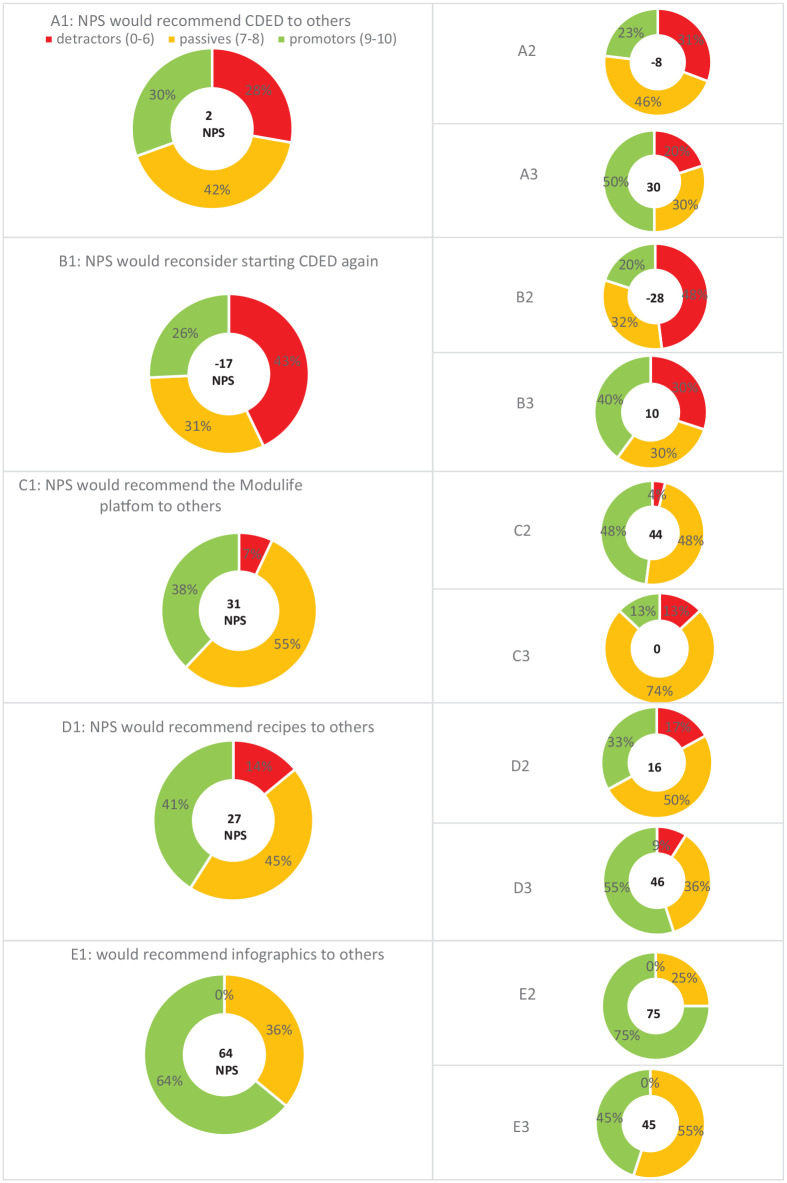
NPS scores regarding CDED. A, recommend CDED to others (A1 all participants (*N* = 36), A2 pediatrics (*N* = 26), A3 adults (*N* = 10)); B, Reconsider starting CDED again (B1 all participants (*N* = 35), B2 pediatrics (*N* = 25), B3 adults (*N* = 10)); C, Recommend the Modulife platform to others (C1 all participants (*N* = 29), C2 pediatrics (*N* = 21), C3 adults (*N* = 8)); D, Recommend available recipes to others (D1 all participants (*N* = 29), D2 pediatrics (*N* = 18), D3 adults (*N* = 11)), and E, Recommend infographics to others (E1 all participants (*N* = 25), E2 pediatrics (*N* = 16), E3 adults (*N* = 9)). CDED, Crohn’s disease exclusion diet; NPS, net promotor scores.

Data on the experience with CDED + PEN and the Modulife patient support platform are shown in Table 2. About half of the patients (45%–55%; depending on the phase of the diet) in the prospective cohort, and the majority (83%) of patients in the retrospective cohort, would recommend CDED to others. CDED was rated more positively by adults (positive NPS) than by pediatrics and/or their parents (negative NPS). Two-thirds of the retrospective patients would reconsider starting CDED again if necessary, based on yes/no questions, with an NPS of +2; adults were more positive than pediatric patients. All NPS scores related to CDED experiences and the Modulife support platform are shown in Figure 1.

**Table 2.** Experiences with CDED + PEN and Modulife patient support platform.

Variables	After phase I (W6)	After phase II (W12)	After phase III (W18)	Retrospective
Experience CDED				
Recommend CDED to others % yes (*N*)	45 (*N* = 13)	45 (*N* = 11)	55 (*N* = 7)	83 (*N* = 40)
Reconsider starting CDED again % yes, (*N*)	Non applicable	Non applicable	Non applicable	67 (*N* = 42)
Feasibility at home % yes/(strongly) agree, (*N*)	72 (*N* = 50)	88 (*N* = 51)	74 (*N* = 46)	Non applicable
Feasibility at school/work % yes/(strongly) agree (*N*)	67 (*N* = 51)	44 (*N* = 48)	54 (*N* = 42)	Non applicable
Estimated additional time spent in hours on meal preparation mean ± SD (*N*)	7 ± 6.4 (*N* = 39)	5 ± 3.7 (*N* = 32)	3 ± 2.3 (*N* = 23)	Retrospective data prescribed for each phase of the diet
Diet burden score (0–10) compared to usual diet mean ± SD (*N*)	Non applicable	Non applicable	Non applicable	6.3 ± 2 (*N* = 38)
Family members’ participation in CDED % ate same meals (*N*)	Non applicable	Non applicable	Non applicable	48 (*N* = 42)
Modulen IBD^®^				
Flavor of Modulen IBD (*N*)	(*N* = 13)	(*N* = 11)	(*N* = 7)	(*N* = 40)
% Very tasty	0	0	0	5
% Tasty	38	27	29	8
% Neutral	23	36	43	28
% Not tasty	8	18	29	23
% Not tasty at all	31	18	0	38
Used Modulen IBD as prescribed (*N*)	(*N* = 13)	(*N* = 11)	(*N* = 6)	(*N* = 42)
% Yes	46	73	83	50
% No, used more	8	0	0	12
% No, used less	46	27	17	38
Modulife patient support platform				
Frequency of use of Modulife platform (*N*)	Non applicable	Non applicable	Non applicable	(*N* = 43)
% Always				25
% Often				49
% Sometimes				14
% Rarely				0
% Never				12
Sufficient support from the platform % yes/(strongly) agree (*N*)	100 (*N* = 13)	100 (*N* = 15)	100 (*N* = 7)	93 (*N* = 41)
Used parts Modulife platform (%)	Non applicable	Non applicable	Non applicable	(*N* = 43)
Asking questions				7
Infographics with diet information				37
Weekly menus				7
Recipes				87

CDED, Crohn’s disease exclusion diet; PEN, partial enteral nutrition.

Patients assessed the feasibility of the diet at home better than at school or work. About a quarter (21%) of participants mentioned that “it is difficult to take suitable meals to school or work.” Patients found the diet more often feasible at school or work in phase I than in phase II. Reasons cited included that “it became more difficult to maintain” and one participant noted that “it was a difficult period with many parties/eating out.” Approximately two-thirds (61%) of the family members of pediatric patients in the retrospective cohort also participated in the CDED diet, whereas the majority (82%) of family members of adult patients did not eat the same meals.

The taste of the PEN formula (Modulife IBD) was rated as moderate: 23%–43% of patients were neutral about the taste, and 29%–61% (depending on the phase of the diet) did not like it. However, patients reported that mixing the Modulen IBD powder with fruit to make a smoothie significantly improved the taste. Half to three-quarters of the patients used the Modulen IBD powder in the prescribed amount, with the lowest percentage of complete adherence to the prescribed amount in CDED phase I. Only a few patients reported using more PEN than prescribed.

The majority of patients (75%) reported using the Modulife patient support platform always or often during therapy and strongly agreed or agreed that the platform is a sufficient or essential element of the diet. Some patients found the product overview for each phase of the diet “very clear and specific,” while others thought “it was clear what is not allowed in the diet, but not which foods are allowed to eat.” The recipes feature was the most used and, together with the infographics, also the most appreciated part of the platform. Some patients used the app to add more variety to their meals, while others mentioned that they missed more variation in the recipes.

There were no relevant differences between adults and pediatric patients in the NPS scores for recommending the Modulife platform, recipes, and infographics to others; all scores were positive.

Tips and tricks for following the diet and using the platform are provided in Table 3.

**Table 3.** Tips and tricks for CDED.

Easy-to-use tips
“I used the weekend to prepare meals for the rest of the week.”
“Prepare everything at home, make it in larger quantities and put several portions in the fridge.”
“Shakes are easy to take to school/work.”
“The taste of the shakes gets better when mixed with the fresh fruit.”
“Use the Modulife platform and the recipes!”
Tips for improvement for healthcare professionals
“I missed a list with examples of approved products and where to buy them.”
“I missed background information about why some products are (not) approved.”
“I would like to have a list with ingredients to avoid.”
“I missed a list with products that can be eaten when playing with a friend/staying over, e.g. Matze crackers, egg, apple, banana.”
“I missed tips about where to buy the right rice flour.”
“The recipes could be better adjusted to several food cultures.”
“CDED cooking demos/videos or workshops would be nice and make it easier to make the recipes.”
“I would like to talk about our experiences with the diet with other patients who follow the diet.”
“I would have liked to have more frequent contact with the dietician during the diet.”
“Follow the diet with other persons is supportive and makes it easier to maintain.”
“Check the effect of the diet earlier to not continue a diet unnecessarily for a long time or, when it is effective, as a motivation to continue.”
“Have a test phase of the diet first to see whether the diet is suitable for a patient/whether someone can handle it before you start CDED as long-term treatment.”

CDED, Crohn’s disease exclusion diet.

### CDED compliance

Data on dietary compliance are depicted in Table 4. Three-quarters (73%) of the patients (retrospectively) reported never eating a non-permitted product during phase I, compared to approximately half of the patients (47%) in phase II. None of the patients reported consuming a “cheat” product more often than 3–4 times a week during any phase of the diet. Examples of “cheat” products consumed include “chips, cake/candy, and coffee.”

**Table 4.** How compliant is the patient with the dietary therapy?

Patient compliance	Phase I	Phase II	Phase III
Retrospective^a^ (%)	(*N* = 40)	(*N* = 36)	(*N* = 29)
Good compliant	83	80	70
Poor/non-compliant	17	20	30
Dropout (%)	9% (1 adult/3 children)	26% (3 adults/10 children)	
Prospective^b^ (%)	(*N* = 19)	(*N* = 14)	(*N* = 10)
Good compliant (>10 points)	42	36	40
Moderate compliant (7–10 points)	26	36	30
Poor/non-compliant (<6 points)	0	7	0
Dropout (%)	32% (1 adult/5 children)	21% (2 adults/1 child)	30% (2 adults/1 child)

a Based on consuming cheat products.

b Based on Supplemental Table 1.

The number of dropouts in the prospective study was highest in phase II. Patients who continued phase I until the end were the most likely to be classified as good adherents. Overall, the percentage of patients who continued the diet and were labeled as poor/non-compliant was nearly zero. More detailed outcomes of the MARS questions (1 and 2–10) are depicted separately in Supplemental Figures 1 and 2.

### Effectiveness (physicians’ and patients’ perspectives)

Data on the effectiveness of the dietary therapy are depicted in Table 5. After 18 weeks of CDED + PEN, the median FCP gradually decreased from 1378 to 76 µg/g in the total population, and from 1572 to 84 µg/g in the pediatric population (both *p* < 0.001). CRP followed a similar trend, gradually decreasing over time in both the full cohort and specifically in the pediatric group (both *p* < 0.001). In the adult population, an overall decrease in FCP from 650 to 38 µg/g was observed after 18 weeks, which was preceded by a primary initial increase during the first phase of the diet. The median score subjectively by patients given for the effect of the diet on their complaints was 8 (5–9) (range 0–10), while the median score for experience was 7 (6–7) (range 2–8.5). In addition, the majority of physicians (68%–75%) assessed the diet as showing good effectiveness and would continue the therapy (69%–76%) at each phase. After phases I and II of the diet, 55% of physicians would consider starting additional therapy, which changed to 42% after phase III. In approximately 70% of cases, both patients and physicians were positive about the effect of the dietary therapy.

**Table 5.** Effectiveness of CDED therapy from the perspectives of both physicians and patients.

Variables	Pediatric	Adult	Total
FCP µg/g, median (IQR) (*N*)			
Start CDED/baseline	1572 (611–3047) (45)	650 (33–2007) (13)	1378 (505–2900) (58)
End phase I (W6)	867 (173–2960) (35)	3461 (1768–4392) (6)	1000 (198–3114) (41)
End phase II (W12)	388 (92–850) (27)	2664 (757–5458) (4)	441 (110–1162) (31)
6 weeks after phase III (W18)	82 (30–490) (29)	38 (9–2973) (8)	70 (24–628) (37)
Difference baseline-W18	*p* < 0.001	*p* = 0.753	*p* < 0.001
CRP mg/L, median (IQR) (*N*)			
Start CDED/baseline	6 (1–30) (44)	3 (1–40) (13)	6 (1–32) (57)
End phase I (W6)	2 (1–6) (35)	6 (2–60) (4)	2 (1–6) (39)
End phase II (W12)	1 (1–8) (30)	6 (0–32) (3)	1 (0–8) (33)
6 weeks after phase III (W18)	1 (1–3) (32)	4 (2–6) (5)	1 (0–4) (37)
Difference baseline-W18	*p* < 0.001	*p* = 0.593	*p* < 0.001
Effectiveness assessed by physician % yes (*N*)			
End phase I (W6)	74% (27)	50% (10)	68% (37)
End phase II (W12)	83% (23)	43% (7)	73% (30)
6 Weeks after phase III (W18)	77% (22)	67% (6)	75% (28)
Effectiveness assessed by the patient % yes	*N* = 31	*N* = 11	*N* = 42
End phase I (W6)	Non applicable	Non applicable	Non applicable
End phase II (W12)	Non applicable	Non applicable	Non applicable
6 Weeks after phase III (W18)	74%	73%	74%
Agreement on the judgment of effectiveness of therapy: physician vs patient	*N* = 21	*N* = 6	*N* = 27
% Both assessed as effective	71	67	70
% Effective according to physician, not patient	10	0	7
% Effective according to patient, not physician	14	33	19
% Both were assessed as not effective	5	0	4

CDED, Crohn’s disease exclusion diet; CRP, C-reactive protein; FCP, fecal calprotectin; IQR, interquartile range.

## Discussion

This is the first study reporting on real-life data on patients’ experiences and perspectives of dietary therapy CDED + PEN in mild-to-moderate active CD patients. We showed that the majority of patients who followed the CDED are satisfied with the therapy and its effect and have useful information to share with prescribers and other patients. These patients scored high at recommending CDED to others, reconsidering starting the diet again (if necessary), and the usefulness of the accompanying patient support platform. Two-thirds of the patients as well as physicians considered the therapy effective.

As adherence is a key factor in the effectiveness of dietary therapy, dieticians play a crucial role in instructing, motivating, guiding, and monitoring the patient.^18,30–32^ Previous research has shown that compliance in CDED is necessary for ongoing effect.^5,37^ The interviews performed in this study have provided many practical tips that can be incorporated into the treatment.

Implementing CDED + PEN is challenging. It requires meal prepping, contains new and time-consuming recipes, and when PEN additional modification into smoothies is often required to increase adherence.^32^ Our survey showed that most patients considered the diet feasible. The majority (72%–88%) of the patients who have completed the therapy reported the diet to be feasible at home and even 44%–67% feasible at work or school. Interestingly, our survey showed that most patients considered the diet feasible. The majority (72%–88%) of the patients who have completed the therapy reported the diet to be feasible at home and even 44%–67% feasible at work or school.

A dropout rate of 9% in phase I and 26% in phase II was observed due to difficulties with the diet and/or worsening symptoms or the need for medication in our cohorts. It seems that patients who remain on the diet until week 18 are highly compliant and motivated to follow dietary instructions. The positive reported experiences indicated that dietary responders are also the most positive ones and willing to continue.

With regard to starting dietary therapy in children, our data emphasize the importance of assessing the patient’s family situation and social settings to address possible adherence issues early on.^32^

Measuring dietary adherence or compliance is difficult and is performed in different ways or not explained in previous nutritional intervention studies since no specific and validated tool is available.^18,37^ This makes comparison of the compliance data between the current and available studies impossible. Recent attempts have been made to develop tools that can unambiguously measure dietary compliance.^38^

A good biochemical response (e.g., CRP and FCP) was seen in pediatric and adult patients at week 18 versus baseline when therapy was completed. In these patients with a diet-responsive phenotype (as demonstrated by a clinical response during the first two phases of CDED), good adherence was reported. However, caution is needed when interpreting these results, because a part of all patients (20%) started the diet in combination with medication as induction therapy. In the small adult cohort, although the biochemical response lagged, as seen in an increase in FCP after phase I, adults reported feeling better. Adult patients who experienced an increase in FCP during the diet were those who had previously failed multiple treatments (*N* = 3) or had a severe disease phenotype (*N* = 1). These patients showed good compliance and evaluated the CDED therapy even more positively than pediatric patients. We have previously shown that the microbiome/metabolome shifts associated with dietary therapy occur as treatment weeks progress.^39^

Our study followed patients up until 6 weeks after the start of phase III of the diet. There is only limited experience with CDED phase III: the study by Yanai et al. reported favorable rates of mucosal healing in patients continuing the diet and the recently completed DIETOMICS trial^40^ showed the ability of dietary therapy (in combination with immunomodulators for some after EEN induction but also as dietary monotherapy for some patients continuing CDED phase III).^23^ We and others have shown a rebound of calprotectin (in some accompanied by clinical symptoms) when food items are reintroduced after the initial phase of dietary therapy.^15,41^ Despite initial biochemical responses in earlier phases, phase III involves the reintroducing of a broader range of foods over the long term, which may inadvertently trigger inflammatory responses. Moreover, the variability in individual responses underscores the complexity of managing CD, suggesting that a one-size-fits-all approach may be inadequate. Further research is needed to tailor dietary interventions more effectively and improve patient outcomes.

Recent literature in children shows that we can predict response to EEN by identifying dietary patterns, microbial signals, and diet-related metabolites in feces.^39,42^

In adults, CDED was used significantly more as an add-on therapy to continuing medical therapy (*p* < 0.05). This was partly due to the fact that the CDED + PEN index RCT had not been published yet when they got their CD diagnosis. Our data add to the existing cohort studies showing the benefit of dietary management to improve response to medical therapy and subsequent lifestyle modification to maintain response to this novel combination therapy.^19,32,43–49^ In contrast with EEN, where there is an increase in calprotectin when patients switch to a free diet, CDED offers a structure for adding several ingredients with frequent checks of calprotectin.^41,50^ In contrast with EEN, where there is an increase in calprotectin when patients switch to a free diet, CDED offers a structure for adding several ingredients with frequent checks of calprotectin.^41,50^

PEN plays an important part in both the effectiveness of the CDED and the nutritional completeness of the diet.^18^ Compliance with PEN intake is therefore important, and palatability is an essential aspect to ensure adequate intake. In this study, approximately one-third of the patients did not like the taste and flavor of the provided formula (Modulen IBD) and about one-third used less than prescribed amounts, with a risk of inadequate intake. In the era of EEN, before CDED, the palatability of the provided formula was also often raised as a problem contributing to dropout or a switch to nasogastric tube feeding.^4^ The patients included in the current cohorts reported clearly that formula tolerability improved significantly with adding fresh fruit, aided by the available recipes with condiments (herbs and spices) listed on the support platform. It may be considered to use an alternative formula, which is both effective and commonly used in EEN, to increase tolerance and compliance with partial enteral nutrition (PEN) intake. The role of dieticians is pivotal because they can help with cooking tips, recipes, eating out, school, work, vacations, and so on. This creates a more personalized approach and adjustments for patients following the CDED, which can change the adherence to the diet in both children and adults.^32^

Combining two cohorts with different data collection methods can be challenging to interpret due to bias in retrospective cohorts, while we acknowledge the bias introduced by combining two cohorts, we used almost identical data collection methods and aligned patient experiences with clinical outcomes and adherence. Limitations of the present study were the relatively small number of patients (particularly adults) and the partially retrospective character of the study, which also led to missing values on some of the secondary outcome measures. In the retrospective cohort, interviews were performed after finishing the therapy. It is possible that the results are overestimated since the interview response rate was 50%. It is also possible that patients with a more positive attitude toward nutritional therapy were more inclined or willing to participate. However, this study design enabled a larger cohort of children and adults treated with CDED + PEN to be included, and participants appreciated being asked about their opinion on the effect, performance, and implementation of the diet and provided tips and tricks to help others, as was the case in the early days of the development of this novel dietary approach.

## Conclusion

To conclude, it seems that many mild-to-moderate active CD patients may experience positive outcomes and have good experiences with the CDED + PEN dietary therapy and the associated Modulife patient support platform. They might recommend the therapy to others and consider restarting the diet if necessary. Maintaining good adherence to CDED appears to be essential for effectiveness, in line with the efficacy data from the index RCTs. CDED could be considered as an add-on (“dietary combination”) therapy with favorable clinical and calprotectin response in addition to medical therapy with the option of continuing dietary management during phases II–III in patients who responded clinically in the first phase. Our report adds valuable patient perspectives to the growing clinical use of CDED in managing CD.

## Supplemental Material

sj-docx-1-tag-10.1177_17562848251323553 – Supplemental material for Patient experiences with and adherence to Crohn’s disease exclusion diet in Dutch Crohn’s disease patients: a cohort study Supplemental material, sj-docx-1-tag-10.1177_17562848251323553 for Patient experiences with and adherence to Crohn’s disease exclusion diet in Dutch Crohn’s disease patients: a cohort study by Fleur T. R. Wijers, Suzanne M. C. van Zundert, Charlotte M. Verburgt, Nikki van der Kruk, Johan E. Van Limbergen and Nicolette J. Wierdsma in Therapeutic Advances in Gastroenterology

## Appendix

### Abbreviations

BMI body mass index

BSE blood sediment

CD Crohn’s disease

CDED  Crohn’s disease exclusion diet

CDED + PEN Crohn’s disease exclusion diet plus partial enteral nutrition

CRP C-reactive protein

EEN exclusive enteral nutrition

FCP fecal calprotectin

IBD inflammatory bowel diseases

MARS Medication Adherence Report Scale

MRI magnetic resonance imaging

NPS net promotor scores

## Declarations

**Ethics approval and consent to participate:** April 8, 2021 (reference number W21_187 # 21.202).

**Consent for publication:** We confirm that all necessary consents for publication have been obtained. This includes consent from all individuals mentioned in the manuscript, as well as approval for any data or images included. We have ensured that all ethical guidelines and privacy considerations have been met in accordance with institutional and journal policies.

**Availability of data and materials:** The data supporting the findings of this study are available upon reasonable request. All relevant datasets, including raw data and Supplemental Materials, can be accessed by contacting the corresponding author.
